# *Pearsonema (syn Capillaria) plica *associated cystitis in a Fennoscandian arctic fox (*Vulpes lagopus*: a case report

**DOI:** 10.1186/1751-0147-52-39

**Published:** 2010-06-12

**Authors:** Xavier Fernández-Aguilar, Roland Mattsson, Tomas Meijer, Eva Osterman-Lind, Dolores Gavier-Widén

**Affiliations:** 1 Department of Pathology and Wildlife Diseases. National Veterinary Institute (SVA), SE-751 89 Uppsala, Sweden; 2 Department of Zoology, Stockholm University, 106 91 Stockholm, Sweden; 3 Department of Virology, Immunobiology and Parasitology. National Veterinary Institute (SVA), SE-751 89 Uppsala, Sweden; 4 Department of Biomedical Sciences and Veterinary Public Health, University of Agricultural Sciences (SLU), SE-751 89 Uppsala, Sweden

## Abstract

The bladderworm *Pearsonema (syn Capillaria) plica *affects domestic dogs and wild carnivores worldwide. A high prevalence in red foxes (*Vulpes vulpes*) has been reported in many European countries. *P. plica *inhabits the lower urinary tract and is considered to be of low pathogenic significance in dogs mostly causing asymptomatic infections. However, a higher level of pathogenicity has been reported in foxes. A severe cystitis associated with numerous bladderworms was found in a captive arctic fox (*Vulpes lagopus*) originating from the endangered Fennoscandian arctic fox population. To our knowledge this is the first description of *P. plica *infection in an arctic fox.

## Background

The arctic fox (*Vulpes lagopus; *syn. *Alopex lagopus*) inhabiting the mountain tundra of Fennoscandia (Norway, Sweden and Finland) is regionally threatened to extinction [1]. The main threats today are inter-specific competition with red foxes (*Vulpes vulpes*) and low food availability in certain periods due to the lemming (*Lemmus lemmus*) cycle [2]. In addition, a small population size in combination with a fragmented structure may convey negative effects related to the smallness itself [3]. As part of the conservation efforts, a breeding-in-captivity program was initiated in 1992 at a Swedish breeding center for endangered species ("The Nordic Ark"). The program produced offspring but the foxes were affected by a neurological disease which resulted in the termination of the breeding colony [4]. The foxes of the colony were closely monitored for diseases and thorough post-mortem investigations were conducted on the animals that succumbed. The necropsy in one of the arctic foxes revealed numerous worms in the urinary bladder identified as *Pearsonema plica*. A severe cystitis was associated with the presence of the parasites.

*Pearsonema plica *(Syn. *Capillaria plica*) belongs to the superfamily Trichuroidea and inhabits the urinary bladder of the host. The ureters and the renal pelvis are however rarely affected. The infection has been described in domestic dogs and wild carnivore mammals worldwide and occurs frequently in some species [5-8]. The prevalence varies according to hosts, geographic region and season. Several studies have revealed a high prevalence in red foxes in many European countries [8-10].

*P. plica *has an indirect life cycle. The eggs are passed out with the urine from the definitive host and are ingested by earthworms. In the intestine of the earthworm, the eggs hatch and the first stage larvae move through the intestinal wall to encyst in the adjacent connective tissue. Once the earthworm is eaten by the definitive host, the first stage larvae moult to the second stage within the wall of the small intestine. It remains there from day 8 to day 10 after invasion. Third stage larvae are found in the urinary bladder on or about the 30^th ^day of infection and it is assumed that they migrate through the blood vessels to the urinary bladder. The development to the fourth stage larvae and adult worm takes place within the bladder. The prepatent period is around 8 weeks. Besides the young carnivores, adult carnivores are also infected with no evidence of developing acquired immunity. Nonetheless, the infection appears to be self-limiting with a reduction of egg excretion until being undetectable after around 2 1/2 months [11,12].

*P. plica *is considered to be of low pathogenic significance and in most cases, the parasite establish only asymptomatic infections [13-16]. Occasionally cystitis and secondary bacterial infections occur. Such cases have been described in domestic dogs showing dysuria, hematuria, pollakiuria, polydipsia and urinary incontinence [12,17-19]. In farmed silver foxes [18,19] and in experimentally infected red foxes [11] anorexia, dysuria, delayed growth and abnormality in mating have been reported.

To our knowledge, *P. plica *has not been reported previously in the arctic fox. This study describes the presence of *P. plica *and associated lesions in a young arctic fox and the pathogenic potential of this parasite in wild carnivores is discussed.

## Case presentation

The case of this study was a juvenile female arctic fox born in captivity at The Nordic Ark in a litter of 4 cubs, whose dam had been caught in the wild. The dam and the four cubs showed dry cough and mild respiratory signs about three months before developing neurological signs due to necrotizing encephalitis as described previously in arctic foxes [4]. The fox had been treated with B-vitamins, antimicrobial drugs and corticosteroids. No improvement was observed and the fox was euthanized at 7 months of age. Another cub in the litter was euthanized one month later.

Blood samples were obtained just before euthanasia for serology, hematology and clinical chemistry. A post-mortem examination was conducted and specimens of all major organs were sampled for histopathology, fixed in 10% neutral buffered formalin, processed and embedded in paraffin. Sections were cut at 5 μm and stained with hematoxylin and eosin. Selected sections from the urinary bladder and kidneys were also stained with Van Gieson's stain for connective tissue and with periodic acid-Schiff for glycogen and other carbohydrates. Intestines, lung, muscle tissue and urinary bladder were examined for parasites by direct microscopy, the intestinal contents by a sedimentation method, and muscle for *Trichinella *spp by the digestion method. Serological analyses were conducted for antibodies against *Encephalitozoon cuniculi, Toxoplasma gondii, Neospora caninum*, distemper virus and contagious canine hepatitis. Samples for virus isolation in A-72 cells included lungs, liver, kidney, brain and spleen [4].

At post-mortem examination the fox was found in good body condition and weighed 3.8 kg. Multiple areas of diffuse haemorrhage on the surface of the kidneys and a hyperemic mucosa in the urinary bladder were observed. About 20 threadlike, 2-3 cm long nematodes were found in the bladder. According to their morphology and localization, the parasites were identified as *P. plica*. Examination of faeces revealed the presence of eggs of Eucoleus (syn Capillaria) spp. These were probably eggs of *Eucoleus *(syn. *Capillaria*) *aerophilus *subsequently found histologically in the lung and upper respiratory airways.

**Figure 1:**
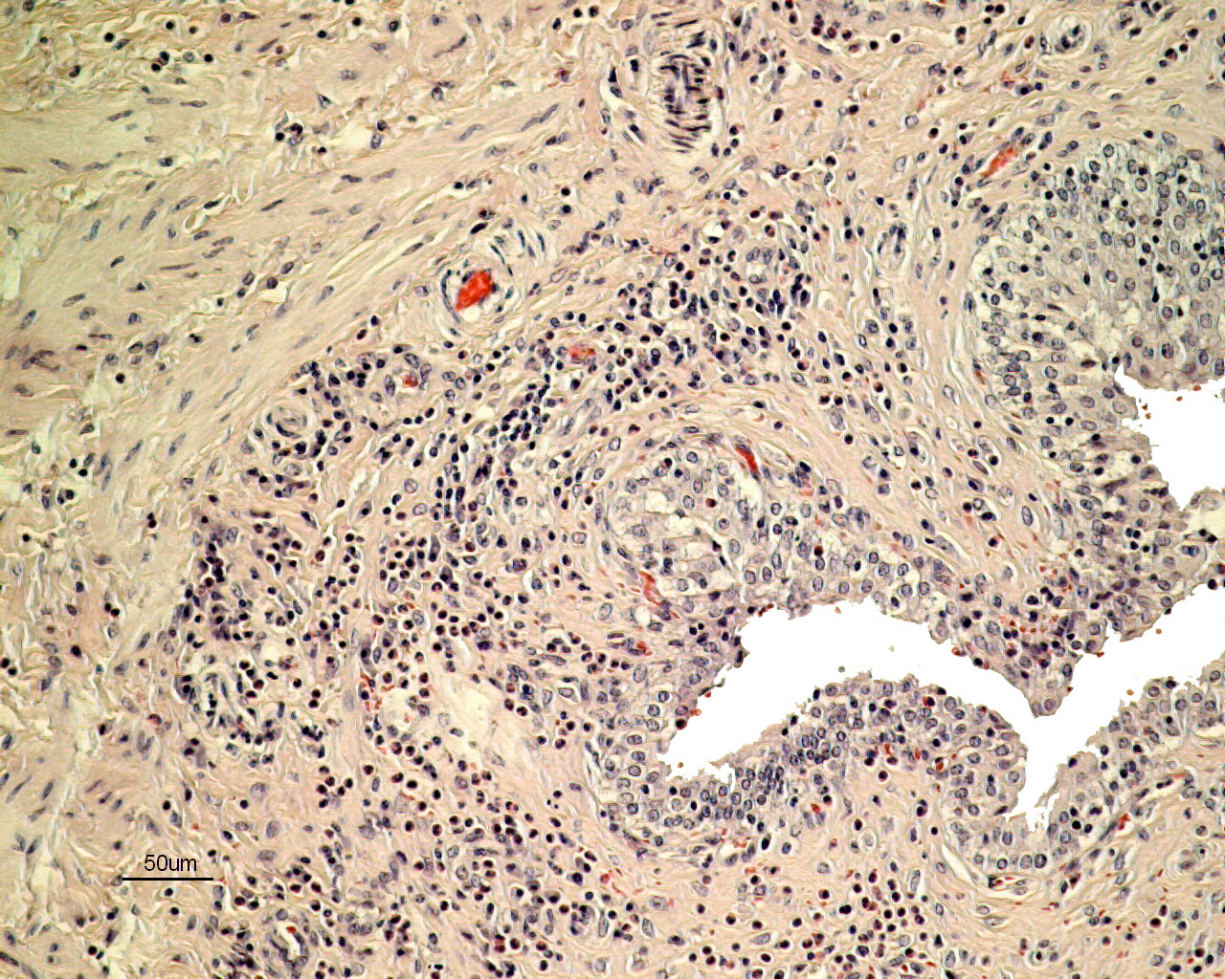
**Histopathology of the urinary bladder of an arctic fox with *Pearsonema plica *infection**. Severe inflammatory infiltration of predominately eosinophils in the lamina propria and in superficial layers of the muscularis propria. Stain: hematoxylin and eosin.

**Figure 2:**
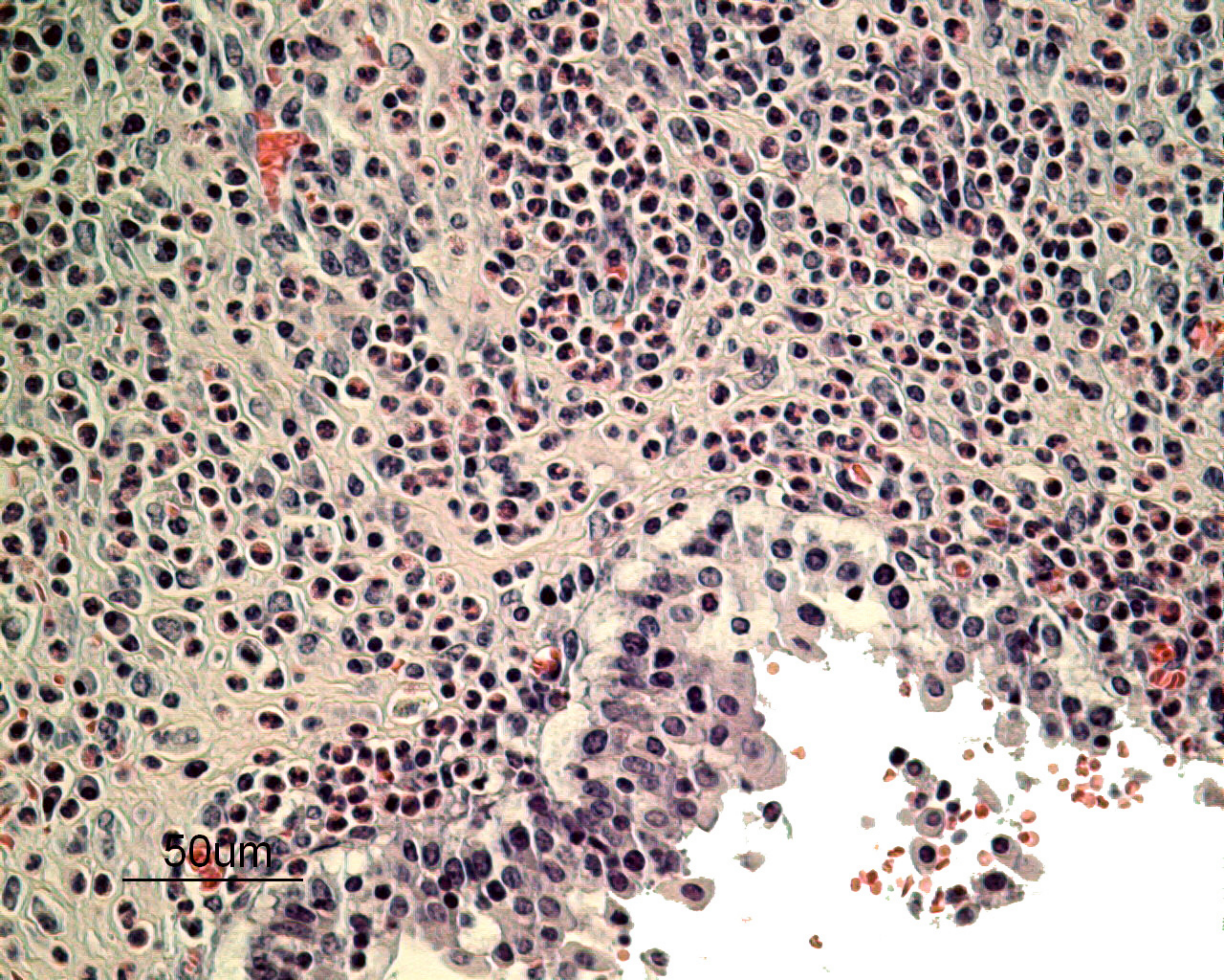
**Histopathology of the urinary bladder of an arctic fox with *Pearsonema plica *infection**. Dense infiltrate of eosinophils in the lamina propria, degeneration and detachment of urothelium and microhaemorrhages. Stain: hematoxylin and eosin.

**Figure 3:**
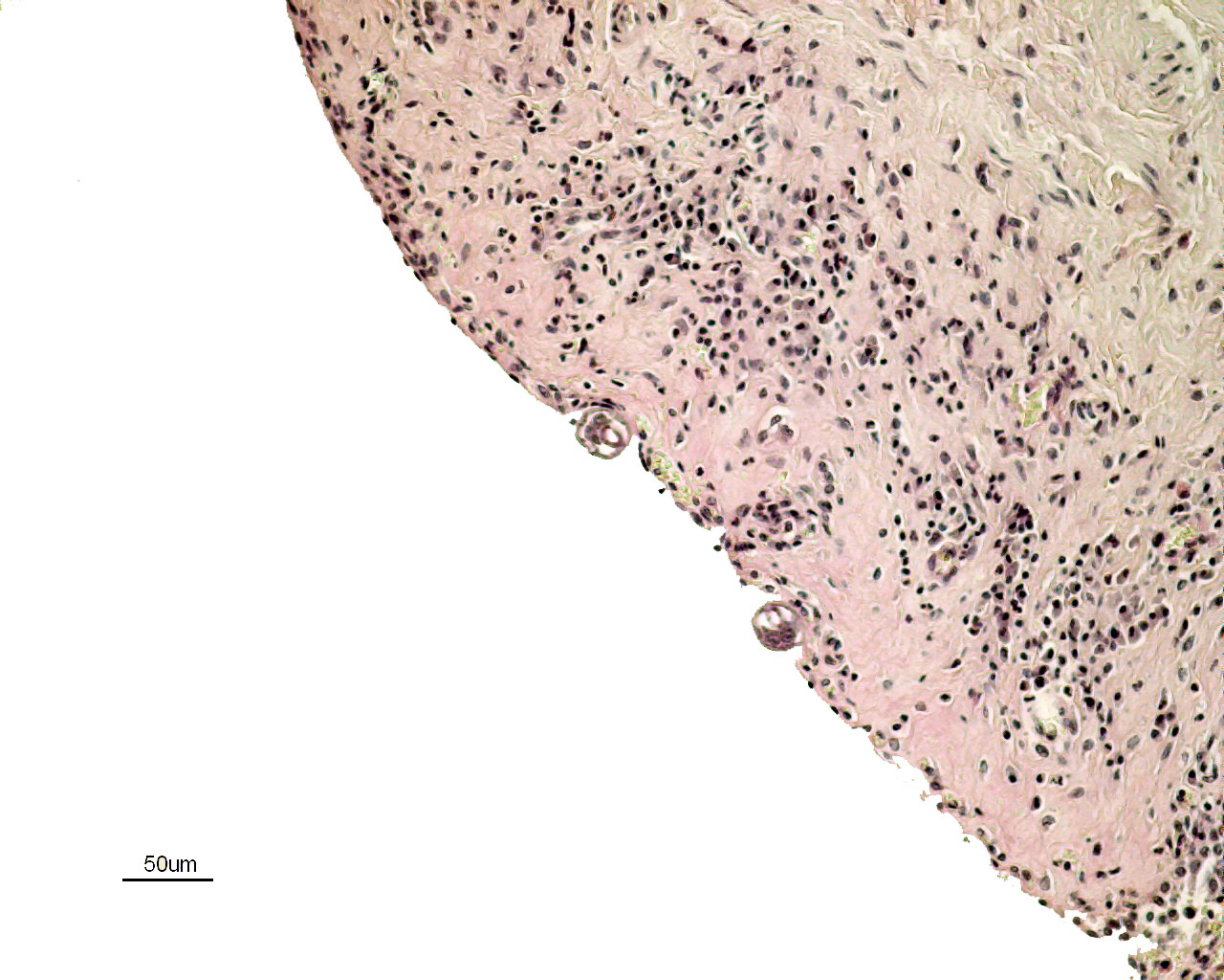
**Sections of *Pearsonema plica *in the urinary bladder of an arctic fox**. Sections of the parasite embedded in the wall of the bladder. Stain: hematoxylin and eosin.

**Figure 4:**
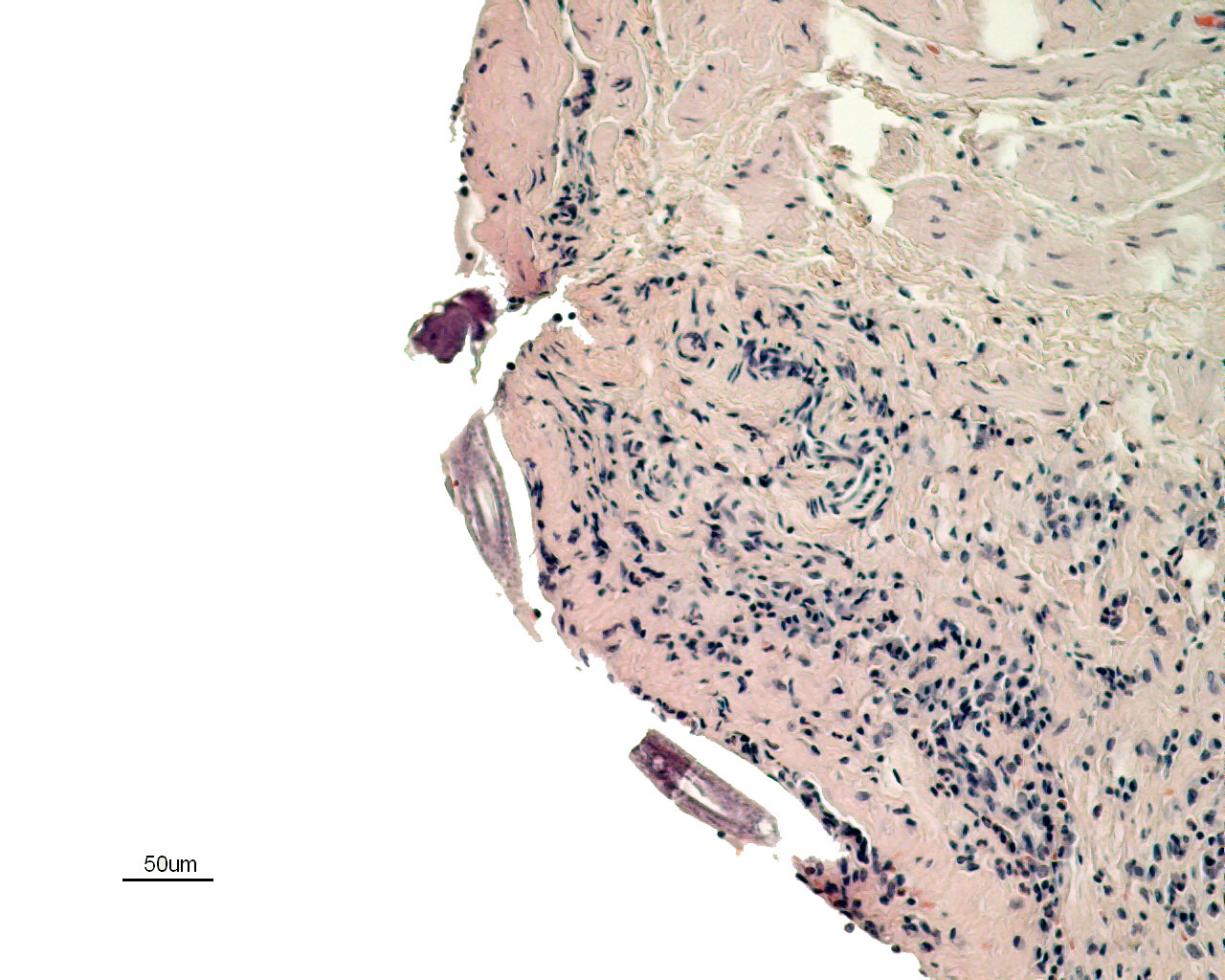
**Sections of *Pearsonema plica *in the urinary bladder of an arctic fox**. The rostral end and the body of the parasite are embedded in the wall of the bladder. Stain: hematoxylin and eosin.

The histopathology of the urinary bladder showed a severe sub-acute inflammation with infiltration of predominantly eosinophils with some plasma cells and lymphocytes in the mucosa extending into the inner muscular layers (Figure 1). The urothelium showed intraepithelial infiltration of eosinophils, degeneration and detachment. There was congestion of the mucosa and small haemorrhages in areas subjacent to the epithelial damage (Figure 2). There was apparent fibroplasia in superficial parts of the lamina propria with a severe inflammatory infiltrate. Sections of nematodes were present in the lumen of the bladder adjacent to the surface (Figure 3 & Figure 4). The rostral end of a worm was seen embedded into the superficial mucosa. In the kidneys, infiltration of few mononuclear cells and eosinophils in the submucosa of the pelvis was seen. The lungs presented interstitial, eosinophilic pneumonia with granulomatous reactions around *Capillaria *spp eggs. In the trachea there was also a moderate eosinophilic infiltration comprising the mucosa and submucosa with exudate and debris in the lumen. The fox also had a severe non-suppurative meningoencephalitis as described in other cases [4].

Blood parameters, hematocrit, urea, creatinine, aspartate aminotranseferase (ASAT), creatine kinase (CK), alanine aminotransferase (ALAT), alkaline phosphatase (ALP) and albumin were within the normal range for dog. Reference values for the arctic fox are not available. The number of eosinophils was slightly increased (18% - 1,5×10^9^/L) as were lymphocyte numbers (38% - 3,2×10^9^/L), while neutrophil numbers were diminished (36% - 3,1×10^9^/L). The total leukocyte count remained within the normal range. The other analyses did not reveal abnormalities or signs of infections.

To our knowledge, *P. plica *infection has not been reported before in arctic foxes. For endangered species, it is particularly important to identify and catalogue the pathogens which may affect their survival thus allowing monitoring and, when suitable, intervention. This is particularly true for hosts exposed to long periods of starvation, concomitant parasitic and/or other infections and environmental stress, such as it is often the case of the Fennoscandian arctic fox in the wild. Even though the fox of this study was not free-ranging and the source of infection remains unknown the finding of *P. plica *related cystitis demonstrates that this nematode may cause disease in the arctic fox and therefore, this infection should be included in the health monitoring of the endangered Fennoscandian arctic fox. Moreover, studies that investigated specifically the occurrence of *P. plica *found a high prevalence in red foxes in other regions of Europe and *P. plica *seems to be a common parasite in this species [8-10]. The inter-specific increasing competition between arctic foxes and sympatric red foxes [2] may impact in the parasites presence and dynamics in both populations, including the possible prevalence of *P. plica *in free ranging arctic foxes, and could be considered as a risk factor for the endangered population.

Information about the pathogenicity of *P. plica *varies. Mostly based on studies in dogs, it seems commonly accepted, that the parasite only causes subclinical infection and is of low pathogenic significance [13-16]. However, earlier studies reports a higher level of pathogenicity in foxes [11]. Red foxes were observed suffering of chronic cystitis with signs of anorexia, hematuria, dysuria and delayed growth. Silver foxes showed severe cystitis with red, thickened, swollen mucosa of the bladder and abnormality in mating procedures [18,19]. In the case of this study, clinical signs of cystitis were not observed, but possibly they were masked by the more severe signs of encephalitis. Regardless, signs of cystitis in wild animals are often overlooked. Most authors consider that the cystitis related to *P. plica *is related to secondary bacterial infections. In our case, the fox had received antibiotic therapy and there was no histological indication of secondary infection. It appeared histologically that the parasite itself was damaging the mucosa of the bladder. Eosinophilic inflammation was also observed in the renal pelvis. These observations agree with a previous report, which describes severe damage to the urinary tract [12]. The findings in this case indicates that at least under special circumstances, such as in animals with concomitant diseases or subjected to corticosteroids treatment, the pathogenicity of *P. plica *may be higher than generally recognized.

Besides the case in the fox described here, another juvenile arctic fox from the same litter, which had a similar clinical history and was euthanized at the age of 8 months, also had severe sub-acute eosinophilic inflammation of the urinary system with damage of the urinary bladder mucosa. The renal pelvis showed more severe and extensive infiltrate of eosinophils than the present case. However, the presence of *P. plica *could not be confirmed in this latter fox as parasitological investigation of the bladder was not conducted. Even though infestations of this parasite are usually self-limiting, treatment of captive arctic foxes with clinical disease may be necessary. A single subcutaneous dose of ivermectin (0.2 mg/kg) has been reported to be effective in domestic dogs and cats [20,21] and might also be effective in foxes. Routine post-mortem examination of wild animals does not usually include examination with magnification or scrapping of the urinary mucosa to detect parasite eggs or adult parasites. The adult *P. plica *is thin, threadlike, and barely visible and therefore difficult to detect unless performing microscopy. In addition, infected animals do not usually show gross lesions in the urinary tract [12]. These factors may contribute to under-reporting the prevalence of *P. plica *infection in wild carnivores.

## Conclusion

To our knowledge, *P. plica *infection has not been described in arctic foxes. Little is known regarding pathogens in the endangered population of Fennoscandian arctic foxes. The findings of a cystitis related to *P. plica *suggests that in certain cases this nematode may have higher potential pathogenicity in the arctic fox than that generally described in dogs. This is in agreement with early descriptions of *P. plica *infection in foxes

## Competing interests

The authors declare that they have no competing interests.

## Authors' contributions

XFA carried out the pathology investigations, reviewed the literature and drafted the manuscript. EO-L reviewed and discussed the parasitology. RM contributed collecting the data, interpreting the findings and revising the manuscript. TM provided the information on the biology and status of the arctic foxes and revised the manuscript. DG-W coordinated the work of the study, contributed to the pathology investigation and reviewed the manuscript. All authors read and approved the final manuscript.
